# *Entamoeba histolytica *Phosphoserine aminotransferase (EhPSAT): insights into the structure-function relationship

**DOI:** 10.1186/1756-0500-3-52

**Published:** 2010-03-03

**Authors:** Vibhor Mishra, Vahab Ali, Tomoyoshi Nozaki, Vinod Bhakuni

**Affiliations:** 1 Division of Molecular and Structural Biology, Central Drug Research Institute, Chattar Manzil Palace, Council of scientific and industrial research (CSIR), Lucknow 226001, India; 2 Department of Biochemistry, Rajendra Memorial Research Institute of Medical Sciences, AgamKuan, Patna 800007, India; 3 Department of Parasitology, National Institute of Infectious diseases, 1-23-1 Toyama, Shinjuku-Ku, Tokyo 162-8640, Japan

## Abstract

**Background:**

Presence of phosphorylated Serine biosynthesis pathway upstream to the de novo cysteine biosynthesis pathway makes PSAT a crucial enzyme. Besides this, phoshoserine produced by the enzyme can also be taken up directly by cysteine synthase as a substrate. PSAT is a PLP dependent enzyme where the cofactor serves as an epicenter for functional catalysis with the active site architecture playing crucial role in optimum function of the enzyme.

**Findings:**

EhPSAT is a homodimer of molecular mass 86 kDa. To understand the structural modulations associated with pH dependent changes in functional activity of EhPSAT detailed biophysical studies were carried out. pH alterations had no significant effect on the secondary structure, cofactor orientation and oligomeric configuration of the enzyme however, pH dependent compaction in molecular dimensions was observed. Most interestingly, a direct correlation between pH induced modulation of functional activity and orientation of Trp 101 present in the active site of the enzyme was observed. Sodium halides nullified the pH induced global changes in the enzyme, however differential effect of these salts on the active site microenvironment and functional activity of the enzyme was observed.

**Conclusions:**

The study unequivocally demonstrates that pH induced selective modification of active site microenvironment and not global change in structure or oligomeric status of the enzyme is responsible for the pH dependent change in enzymatic activity of PSAT.

## Background

**Figure 1:**
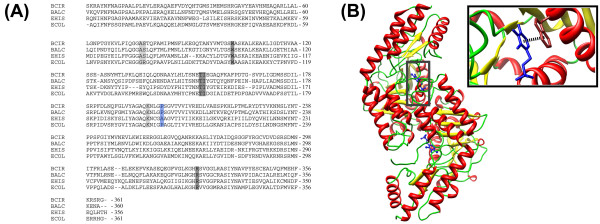
**(A) Alignment of the amino acid sequence of PSAT from *Bacillus circulans *spp. *Alkalophilus *(BCIR), *Bacillus alcalophilus *(BALC), *Entamoeba histolytica *(EHIS), and *E coli *(ECOL)**. Highlighted letters indicate active site residues that interact with the cofactor PLP (light grey) and Cl^- ^ions (dark grey). The substitution of Pro201 (BCIR PSAT) to Ala195 (EhPSAT) in the loop region has been highlighted in blue. (B) Homology model of EhPSAT dimer visualized through UCSF chimera. The cofactor PLP present in the active site is represented in cyan color. The inset shows stacking of the cofactor PLP(cyan color) and the Trp101(brown color) at the active site.

PSAT is a vitamin B_6_-dependent enzyme that belongs to the α-family of pyridoxal-5'-phosphate (PLP) enzymes. It catalyzes the reversible conversion of 3-phosphohydroxypyruvate to L-phosphoserine in a glutamate linked transamination reaction, the second step of phosphorylated serine biosynthetic pathway. Earlier studies on PSAT from *E coli *[1], *Bacillus alcalophilus *[2], *Arabidopsis thaliana *[3], and *Homo sapiens *[4] suggests that the enzyme exist as a homodimer with a subunit molecular mass between 40 to 48 kDa. Each subunit is predominantly composed of two domains, a large PLP binding domain and a small domain comprising of C-terminal part along with a short N-terminal portion [1]. Structurally PSAT is a α/β protein with one PLP molecule present in the active site, per monomer. The dimeric configuration of the enzyme is essential for the functional activity [5]. The active site amino acid residues are nearly conserved in all PSATs (Fig. 1A).

*Entamoeba histolytica *is a protozoan parasite that infects the gastrointestinal tract and causes amoebic colitis and extra intestinal abscesses in humans [6]. Role of L- serine in a number of important metabolic pathways in the parasite has been well established [7,8] Predominantly it serves as a precursor molecule for L-cysteine biosynthesis which plays important role in survival, growth, attachment [9,10], anti-oxidative defense [11], and Fe-S cluster biosynthesis [12]. From the amino acid sequence alignment and phylogenetic analyses EhPSAT shows close association with bacteroide PSAT [13].

The cofactor PLP plays a significant role in structural stabilization of PSAT from extremophiles [14] and serves as a vital tool for understanding the active site stochiometry and conformational rearrangements under various experimental conditions. We have carried out a detailed study on the pH induced changes in the functional, structural and stability properties of EhPSAT and effect of salts on these changes. Additional file 1 carries detailed information regarding the materials and experimental procedures applied in the presented study.

## Results

### EhPSAT production and oligomeric state

**Figure 2:**
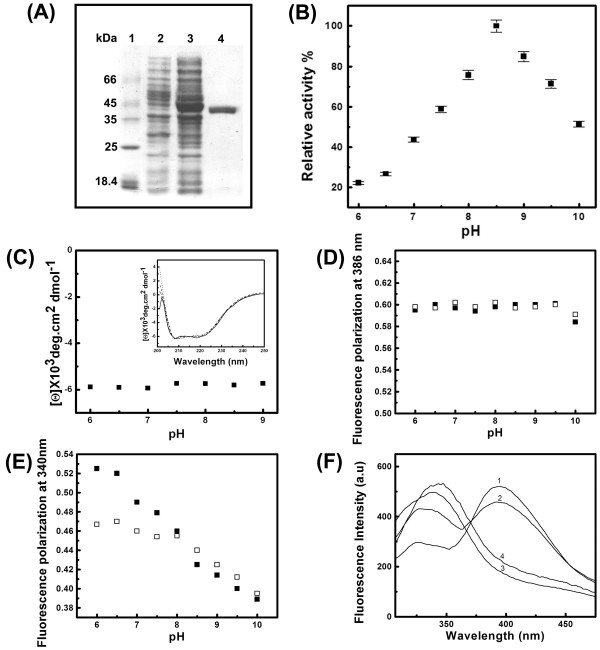
**Overexpression, purification, functional activity and pH dependent structural changes of EhPSAT**. (A) SDS-PAGE analysis of *E coli *lysate over expressing EhPSAT and the purified protein. Lanes 1-4 represent molecular weight markers, uninduced culture, induced culture and purified protein, respectively. (B) pH-dependent enzymatic activity profile of EhPSAT. The data has been represented as percentage relative activity with highest activity observed at pH 8.5 taken as 100%, each point representing mean ± SD of three independent measurements. (C) pH induced changes in the secondary structure of EhPSAT. The effect of pH on the CD signal at 222 nm. The inset shows far-UV CD spectra at pH 6 (solid line), 7 (dashed line), 8 (dotted line) and 9 (dash-dotted line), respectively. (D) and (E) pH-induced changes in PyP and Trp fluorescence polarization, respectively. In both the panels the filled and the open symbols represent protein samples in absence and presence of 200 mM NaCl, respectively. (F) pH induced changes in fluorescence emission spectra of EhPSAT excited at 295 nm. The curves 1-4 represent protein samples incubated at pH 6, 7, 8 and 9, respectively.

**Figure 3:**
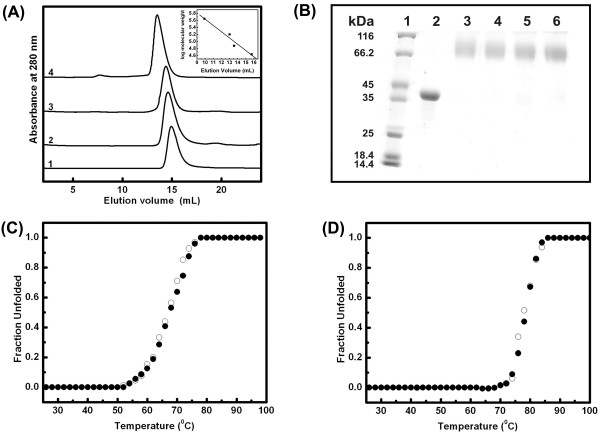
**pH induced compaction of EhPSAT, oligomeric state and thermal stability**. (A) Curves 1-4 represent SEC profile of EhPSAT on a superdex 200, 10/300 GL column at pH 6, 7, 8 and 9, respectively. The inset shows the graph of elution volume plotted against standard molecular mass markers. The proteins are (1) 440 KDa (ferritin), (2)158 KDa (aldolase), (3) 75 KDa (conalbumin) and (4) 43 KDa (ovalbumin). (B) 10% SDS-PAGE profile of glutaraldehyde crosslinked sample of EhPSAT incubated at different pH. Lane 1-6 represent molecular weight markers, uncrosslinked EhPSAT, and glutaraldehyde crosslinked form of EhPSAT at pH 9, 8, 7 and 6, respectively. Cooperative thermal unfolding of EhPSAT at pH 8.5 (panel C) and 6 (panel D) as measured by loss of CD ellepticity at 222 nm and 415 nm. A linear extrapolation of baselines in pre and post transition regions was used to determine the fraction unfolded protein within the transition region by assuming a two state mechanism of unfolding. The open and filled circle represent for far and near UV-CD signals, respectively. The thermal transition of the enzyme was found to be irreversible with precipitation observed at the end of the scan.

The purified protein was homogenous as indicated by a single protein band on SDS-PAGE (Fig. 2A) and a single peak in ESI-MS of molecular mass about 43 kDa (data not shown). The SEC profile of EhPSAT (Fig. 3A) along with glutaraldehyde crosslinking experiments (Fig. 3B) demonstrates that the recombinant EhPSAT is a homodimer under physiological conditions.

### pH dependent changes in functional and structural properties of EhPSAT

#### Functional property

EhPSAT showed pH dependent bell shaped enzymatic activity profile for forward reaction with maximum activity at pH 8.5 (Fig. 2B). At pH 10 and 6.0, the enzyme retained about 50% and 20% residual activity, respectively.

For understanding the structural basis of such a characteristic pH dependence of enzymatic activity, structural changes in the enzyme under these conditions, were studied.

#### Secondary structure

The secondary structure elements are generally conserved among PSAT family of enzymes [1,2]. The secondary structure of EhPSAT as characterized by far-UV CD shows that it is a α/β type protein (Fig. 2C inset). No significant alterations in the CD signal at 222 nm were observed for the enzyme between pH 9 and 6(Fig. 2C). Hence, pH change does not significantly affect the secondary structure of EhPSAT.

#### Active site microenvironment

No significant alteration in fluorescence polarization of PLP was observed between pH 6 to 9 suggesting no change in orientation of cofactor PLP of EhPSAT with change in pH (Fig. 2D). Interestingly for tryptophan residues, with decrease in pH from 10 to 6 an almost linear enhancement in fluorescence polarization was observed, (Fig. 2E). This demonstrates that low pH induces restriction in orientation of tryptophan moiety present in enzyme. EhPSAT contains 3 tryptophan residues, two in the N-terminal region and one, Trp 101, in the active site. We wanted to see whether the above observed changes correspond to the active site tryptophan moiety or not.

On reduction of PLP aldimine, the cofactor serves as a reporter molecule itself and also as an energy acceptor of tryptophan fluorescence provided the two moieties are within a distance of 5 Å [15,16]. For EhPSAT, PLP and Trp 101 are stacked close to each other at the active site (Fig. 1B inset). Fig. 2F summarizes the effect of pH on the fluorescence spectra of EhPSAT on excitation at 295 nm. At pH 8.0 and 9.0, single fluorescence emission maxima at 335 nm and 340 nm, respectively was observed. However, at pH 6 and 7, two clear emission maxima centered at 386 and 335 nm, respectively were observed. Furthermore, on decrease in pH from 9 to 6, a decrease in intensity of signal at 335 nm and a concomitant increase in intensity of signal at 386 nm were observed. These observations demonstrate that between pH 9 and 6 the two fluorophores PLP and Trp101 come closer to each other and show FRET. The FRET studies along with the PLP polarization studies (Fig. 2D to 2F) demonstrate that with change in pH, the orientation of Trp101 residue is modified such that it comes close to PLP cofactor in 3D space.

#### Molecular dimension, Subunit configuration and stability

On decrease in pH from 9 to 6, decrease in the hydrodynamic radii of the enzyme (Fig. 3A) was observed indicating either pH-induced dissociation of native dimer to monomer or compaction of dimeric conformation. Chemical crosslinking is a well accepted technique for studying changes in the oligomeric status of a protein under experimental conditions [16-21]. Fig. 3B shows the SDS PAGE profile of the glutaraldehyde cross linked protein samples. A single species corresponding to dimer of enzyme was observed under all the conditions studied. The stability of dimeric configuration over a broad pH range has also been reported for PSAT from *Bacillus circulans *ssp. *Alkalophilus*. These studies collectively demonstrate that PSAT dimer is stable over a broad pH range.

Fig. 3C and 3D shows thermal denaturation profile of EhPSAT at pH 8.5 and 6 as monitored by the loss of CD signal at 222 and 415 nm. Superimposable far-UV and near UV CD signals demonstrate a high degree of structural cooperativity exist in EhPSAT under these conditions. However, for pH 8.5 and 6, *T*_m _of about 67°C and 79°C, respectively were observed. These obeservations are in agreement with the ProTherm database as reported earlier for *Bacillus circulans *ssp. *Alkalophilus *[14]. Hence, decrease in pH leads to enhanced thermal stability of PSAT.

#### Effect of sodium halides on the enzymatic activity

**Figure 4:**
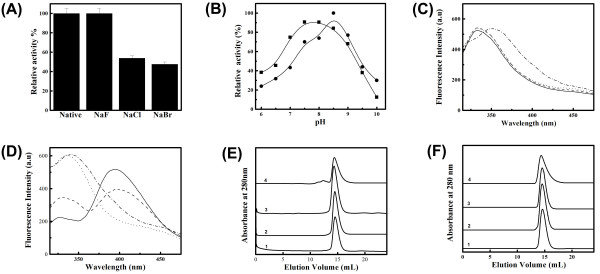
**Effect of Sodium halides on pH dependent changes in functional activity and structural features of EhPSAT**. (A) Inhibition of functional activity in presence of 200 mM concentration of various Sodium halides. The activity of EhPSAT in absence of salt (native protein) at pH 8.5 was taken as 100%. (B) pH dependent enzymatic activity profile of EhPSAT in presence of 200 mM concentration of NaCl (rectangles) and NaF (circles), respectively. For samples in presence of NaCl and NaF the highest value observed was taken as 100%. Fluorescence emission spectra of EhPSAT in presence of 200 mM NaCl (Panel C) and NaF (Panel D) at different pH. The different curves in both the panel represent protein samples at pH 6 (solid line), 7 (dashed line), 8 (dotted line) and 9 (dash dotted line). SEC profile at various pH in presence of NaCl (panel E) and NaF (panel F). The curves 1-4 represent SEC profile in presence of 200 mM concentration of salts at pH 6, 7 and 8, and 9 respectively.

Salts affect the physico-chemical properties of proteins primarily through modulation of electrostatic and hydrophobic interactions acting on the protein molecule. Fig. 4A shows the effect of 200 mM NaF, NaCl and NaBr on the enzymatic activity at pH 8.5. NaCl and NaBr inhibited the enzymatic activity in a concentration dependent manner, while NaF showed no such effect. Detailed studies with NaCl and NaF on pH induced changes in the functional activity (Fig. 4B) clearly show no significant change in the pH dependence of enzymatic activity for NaF. Interestingly, in presence of NaCl the maximum activity was found to be retained over a broader range of pH 7.5 to 8.5 and even at pH 6 about 40% residual activity was observed.

#### Effect of NaCl and NaF on pH induced modification at the active site and strain of internal aldimine of EhPSAT

With change in pH from 8.5 to 6 in the presence of NaCl, no effect on fluorescence polarization of PLP was observed (Fig. 2D). However, the tryptophan fluorescence polarization was found to enhance under these conditions, but to a significantly lower extent as compared to that in absence of NaCl. This demonstrates that the pH-induced change in orientation of tryptophan residue is modulated by the presence of NaCl (Fig. 2E). FRET analyses also support these observations, Fig. 4C and 4D show no significant alteration in the tryptophan microenvironment in presence of NaCl between pH 9 and 6. In contrast for NaF, FRET profile similar to that in absence of salt was observed (Fig. 2F).

The first step in the catalytic mechanism of the enzyme is transfer of a proton of charged substrate amino group to the unprotonated imine nitrogen of the internal Shiff's base in the Michaelis complex. This is followed by nucleophilic attack of the substrate amino group on C4' carbon atom of the cofactor resulting in formation of external aldimine intermediate between PLP and substrate. In PLP catalysis, the electron sink property of cofactor plays an important role. It has been proposed that in the catalytic mechanism the strain and distortion of conjugated π-electron system of PLP and internal aldimine in PSAT are important [22]. When the internal aldimine is protonated a single positive CD band is observed at 410-415 nm but it is shifted to 345-350 nm when the internal aldimine is deprotonated [22]. The additional file 2 shows the pH dependence of near-UV CD spectra of EhPSAT in presence and absence of NaCl. Under both conditions, between pH 6 and 8, a major CD band at 410-415 nm was observed suggesting that the protein is predominantly in protonated state under these conditions. However, for protein samples at pH 9 and 10 the major CD band is shifted to 345-350 nm suggesting that the protein is predominantly in unprotonated state. These observations indicate that the pH- induced difference in the functional activity in absence and presence of NaCl is not due to modulation of pKa of the catalytic reaction.

### Effect of NaCl and NAF on pH-induced compaction and thermal unfolding of EhPSAT

**Figure 5:**
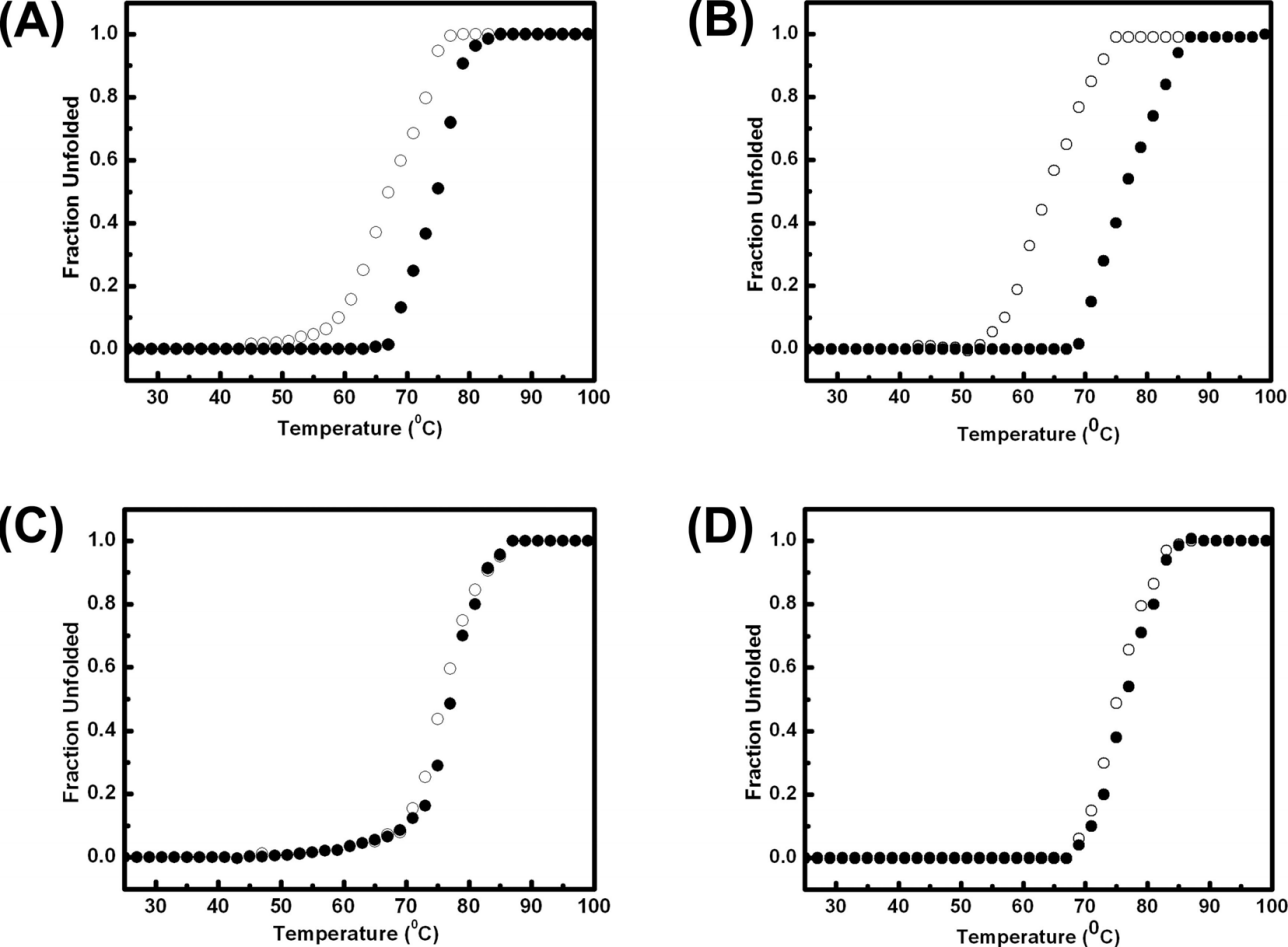
**Thermal unfolding in presence of Sodium halides**. Thermal denaturation profile at pH 8.5 in presence of 200 mM NaCl (panel A), NaF (panel B) and at pH 6 in presence of 200 mM NaCl (panel C) and NaF (panel D), respectively. The open and closed circles represent CD ellepticity measured at 222 nm and 415 nm, respectively. A linear extrapolation of baselines in pre and post transition regions was used to determine the fraction unfolded protein within the transition region by assuming a two state mechanism of unfolding. The thermal transition of the enzyme was found to be irreversible with precipitation observed at the end of the scan.

Fig. 4E and 4F, presents the SEC profile of EhPSAT at pH 6,7,8 and 9 in presence of 200 mM NaCl and NaF, respectively. No significant change in molecular dimension of the protein with change in pH was observed suggesting that pH induced compaction is abolished by salts. Fig. 5A to 5D, shows the thermal unfolding at pH 8.5 and 6.0, respectively in presence of 200 mM NaCl and NaF. At pH 8.5, a significant difference in *Tm *associated with loss of secondary structure and dissociation of PLP from enzyme was observed suggesting the thermal denaturation process in presence of salts to be a noncooperative event. However, at pH 6.0 in presence of salts changes similar to that in absence of salts were observed.

## Discussion

The active site of PSAT is optimized for binding of L-glutamate, 3-phosphohydroxy pyruvate, 2-oxoglutarate and L-phosphoserine [1]. Structural fluctuations in the native state of protein significantly influence the functional activity [23]. The secondary structure of EhPSAT is resistant to pH change between 9 and 6 similar to *Bacillus circulans *spp. *Alkalophilus *(BCIR) PSAT [14]. Hence, the secondary structure of PSATs is stable over a wide pH range and so does not play any significant role in modulation of enzymatic activity.

Crystallographic studies on BALC PSAT clearly suggest the presence of ion binding sites on PSAT molecule[2]. Four Cl^- ^ions in particular are found to be present 2 per monomer close to the active site residue Trp102. Main chain amides of Ser101, Trp102, Thr152 and Ile153 form the binding site for one Cl^- ^ion and the second one is located between positively charged side chain of Arg334 and the N^ε1 ^atom of Trp102(amino acid position described according to BALC PSAT) [2]. Amino acids responsible for chloride ion binding are conserved in BALC PSAT and EhPSAT (Fig. 1A). It is possible that direct binding of Cl^- ^and Br^- ^ions (ionic radii 1.67Å and 1.96 Å, respectively) in the active site leads to structural modifications which results in significant loss of functional activity. F^- ^Ions being smaller in size (ionic radii 1.36 Å) will not bind to the anion binding site(s) with similar affinity and hence not bring about similar change in active site conformation as chloride or bromide ions. This possibility is also supported by the observation that SO_4_^2- ^ions as they also inhibit the enzymatic activity similar to chloride or bromide ions (Data not shown).

The two active sites of PSAT dimer are situated approximately 20 Å apart at the subunit interface. In each active site a PLP molecule is bound with an aldimine linkage to Lys 191 and aditionally hydrogen bonded to residues from the two large domains, besides this prominent stacking interaction at the active site between the pyridine ring of PLP and the indole ring of Trp101, which are usually separated by a distance of approximately 5 Å, occurs on the re-face of the cofactor [[14], this study]. No change in orientation of PLP and tryptophan aromatic ring occurs upon pH change [14]. In case of EhPSAT we observed a pH dependent change in orientation of tryptophan moiety but not for the PLP as monitored by fluorescence polarization (Fig. 2D and 2E). Presence of two fluorophores predominantly stacked with each other in the active site invariably serves as an important tool for probing fine fluctuations of the pH sensitive active site microenvironment. On moving towards low pH (from 8 to 6), the enzyme starts to gradually loose its activity and under these conditions there is a gradual appearance of FRET, suggesting an inwards movement of tryptophan moiety thus bringing it close to PLP in space. In contrast, on moving towards basic pH *i.e*. from 8 towards 10, a shift of tryptophan fluorescence emission maximum from 335 nm to 340 nm was observed, which shows an opening of native conformation of enzyme with partial solvent exposure of tryptophan residue(s). Such pH dependent changes in the active site dynamics suggest relaxation (open) and compaction (close) of active site conformation. Such a dynamic movement in the active site would interfere with proper docking of substrates and responsible for loss of enzymatic activity. Support to such a possibility comes from the pH studies in presence of NaCl where no relative movement of the two fluorophores with pH change (as discussed in paper) was observed and under these conditions no significant alteration in the enzymatic activity was observed over a wider pH range. Conformational changes observed in the present study have not been reported earlier based on the comparison of crystal structures of PSAT at pH 4.6 and pH 8.5[14] because of the fact that protein crystal packing may interfere with fine conformational changes observed in the solution [24,25]. These differences in the active site microenvironment between EhPSAT and BCIR PSAT might probably be due to change in flexibility of the loop region 199-202 which is located near the active site. In this loop the Pro201 in BCIR PSAT is changed to Ala195 in EhPSAT (shown in Fig. 1). Such a change will definitely bring about differences in the flexibility of the loop and subsequently modulate the packing of the protein in the vicinity of active site. The studies presented in the paper clearly demonstrate that local changes at active site microenvironment and not global changes in the protein conformation are responsible for the pH induced modulation of functional activity of PSAT.

## Abbreviations

PSAT: phosphoserine aminotransferase; PLP: pyridoxal-5'-phosphate; Ni-NTA: nickel nitrilotriacetic acid; HPAP: hydroxypyruvic acid phosphate(also called phosphohydroxy pyruvate); SEC: size exclusion chromatography; FRET: fluorescence resonance energy transfer; ESI-MS: electrospray ionization mass spectroscopy.

## Competing interests

The authors declare that they have no competing interests.

## Authors' contributions

VM carried out all the experiments and data analysis. VB concieved and directed the study and was responsible for final evaluation of results. VM and VB drafted the manuscript. VA and TN provided the clone of PSAT and were part of the discussions during drafting of the manuscript. All authors read and approved the final manuscript.

## Supplementary Material

Additional file 1 **Materials and methods**. This file contains information regarding the chemicals, instrumentation and experimental procedures used in the study. This file also describes the methodology implied behind the experimental setup. This file can be opened using Microsoft word. Click here for file

Additional file 2 **Near UV-CD spectra of EhPSAT**. This file contains a figure showing near UV-CD spectra of EhPSAT in absence (Panel A) and presence (Panel B) of 200 mM NaCl. In both the panels the curves 1-4 represent protein samples incubated at pH 6, 7, 8 and 9 respectively. This file can be opened using adobe acrobat reader. Click here for file
