# Supplementary material for: Entamoeba histolytica Phosphoserine aminotransferase (EhPSAT): insights into the structure-function relationship
Source: BMC Res Notes. 2010 Mar 3;3:52. doi: 10.1186/1756-0500-3-52 (PMC2850911; doi:10.1186/1756-0500-3-52)
Supplement: Additional file 1 — Materials and methods. This file contains information regarding the chemicals, instrumentation and experimental procedures used in the study. This file also describes the methodology implied behind the experimental setup. This file can be opened using Microsoft word. [file 1756-0500-3-52-S1.DOC]

**MATERIALS AND METHODS**

**Materials**

All chemicals used in the study were purchased from Sigma-Aldrich chemical company St Louis, USA, and were of highest purity available. Size exclusion chromatographic (SEC) column was purchased from GE Healthcare Bioscience and NiNTA agarose from Quiagen. Milli Q water was used for all experimental work. HPAP was purchased from sigma as the dimethylketal tri(cyclohexylammonium) salt and was converted to HPAP according to the instructions accompanying the reagent.

**Methods**

**Over expression and Purification***-*Recombinant EhPSAT was over expressed in *E.coli* C41 cell. The cells cultured in LB (Luria Bertani) broth containing 100mg/L ampicillin were induced in mid log phase with 0.5mM isopropyl-1-thio-β-D-galactopyranoside and finally incubated at 37ºC for 5 hrs. The cells were harvested at 8000 rpm for 10 minutes and the resultant pellet was resuspended in 50mM potassium phosphate (pH 8) buffer containing 300mM NaCl, 2mM PMSF and 5mM imidazole. Cells were disrupted using a probe type sonicator and centrifuged at 13500 rpm for 20 min. The supernatant was applied on nickel nitrilotriacetic acid (NiNTA) agarose affinity column pre equilibrated with 50mM potassium phosphate (pH 8) buffer along with 300mM NaCl and 5mM imidazole . The column was subsequently washed with the same buffer containing 25mM and 40mM imidazole. The protein was eluted using 400mM imidazole. The eluted protein was tested for purity by SDS-PAGE and ESI-MS and was found to be >95% pure.

**Size exclusion chromatography (SEC)***-* Gel filtration experiments were carried out on a Superdex^TM^ 200,10/300GL column (manufacture’s exclusion limit 600 kDa) on AKTA FPLC (GE Healthcare). The column was pre-equilibrated and run with 10 mM (citrate/ glycine/ hepes) CGH buffer at 25 ºC at a flow rate of 0.3 mL/min, with detection at 280 nm. The column was calibrated with standard molecular weight markers.

**Circular Dichroism (CD) measurements***-* CD measurements were made on JASCO J810 spectropolarimeter calibrated with ammonium (+)-10-camphorsulfonate with 6 µM protein in 10 mM CGH buffer of desired pH with a 2 mm path length cell at 25ºC. The values obtained were normalized by subtracting the baseline recorded for the buffer under similar conditions.

**Fluorescence spectroscopy***-* Fluorescence spectra were recorded with Perkin Elmer LS50B luminescence spectrometer in a quartz cell of 5 mm path length. 6 µM concentration of protein in 10 mM CGH (citrate/glycine/hepes) buffer of desired pH was incubated at 25ºC, before recording the spectra. Excitation wavelength was 295 nm and the spectra were recorded between 300 nm to 500 nm.The reduction of PLP aldimine was achieved using sodium cyanoborohydride according to the procedure described earlier [1].

**Enzyme Activity***-* Recombinant EhPSAT activity was assayed at 25ºC for 5 minutes in 10 mM CGH buffer of desired pH and salt concentrations by method previously described by Ali and Nozaki [2] in a 500 µL reaction mixture, on UV-1650PC, UV-Visible spectrophotometer.

**Cross linking of EhPSAT***-* 50 µg of protein was incubated with 5 µL of 1:25 dilution (w/v) of 25% glutaraldehyde in a 50 µL reaction volume in 10 mM CGH buffer (of desired pH) for 1 hour at 37ºC. The reaction was stopped by adding 5 µL of 1 M Tris- HCl pH 8 in the reaction mixture and the samples were analyzed on 10% SDS-PAGE.

**Thermal denaturation***-* 3 μM and 17 μM concentration of protein samples were subjected to thermal denaturation with change monitored in molar ellipticity at 222 nm or 415 nm respectively, as a function of temperature on a Jasco J810 Spectropolarimeter equipped with peltier temperature controller system. All measurements were performed on protein samples in 10 mM CGH buffer of desired pH at 25 ºC. Samples were heated at constant rate of 1 ºC /min in a 2 mm path length cell.

**Homology modeling and sequence alignment***-* Homology model of EhPSAT homodimer was generated through Swiss Model Server [3] with dimeric crystal structure of PSAT from *Bacillus* spp taken as a template and the image obtained was visualized through UCSF Chimera [4]. The sequence alignments were generated using CLUSTAL W program version 1.83 [5], with blossom 62 matrix.

**REFERENCES**

1. K. Cai and V. Schirch: **Structural studies on folding intermediates of serine hydroxymethyltransferase using fluorescence resonance energy transfer.** *J Biol Chem* 1996, **271:** 27311-20.

2. V. Ali and T. Nozaki: **Biochemical and functional characterization of phosphoserine aminotransferase from Entamoeba histolytica, which possesses both phosphorylated and non-phosphorylated serine metabolic pathways.**  *Mol Biochem Parasitol* 2006, **145:** 71-83.

3. T. Schwede, J. Kopp, N. Guex and M.C. Peitsch: **SWISS-MODEL: An automated protein homology-modeling server.** *Nucleic Acids Res* 2003, **31:** 3381-5.

4. E.F. Pettersen, T.D. Goddard, C.C. Huang, G.S. Couch, D.M. Greenblatt, E.C. Meng and T.E. Ferrin: **UCSF Chimera--a visualization system for exploratory research and analysis.** *J Comput Chem* 2004, **25:** 1605-12.

5. J.D. Thompson, D.G. Higgins and T.J. Gibson: **CLUSTAL W: improving the sensitivity of progressive multiple sequence alignment through sequence weighting, position-specific gap penalties and weight matrix choice.**  *Nucleic Acids Res* 1994, **22:** 4673-80.
