# Supplementary material for: Entamoeba histolytica Phosphoserine aminotransferase (EhPSAT): insights into the structure-function relationship
Source: BMC Res Notes. 2010 Mar 3;3:52. doi: 10.1186/1756-0500-3-52 (PMC2850911; doi:10.1186/1756-0500-3-52)
Supplement: Additional file 2 — Near UV-CD spectra of EhPSAT. This file contains a figure showing near UV-CD spectra of EhPSAT in absence (Panel A) and presence (Panel B) of 200 mM NaCl. In both the panels the curves 1-4 represent protein samples incubated at pH 6, 7, 8 and 9 respectively. This file can be opened using adobe acrobat reader. [file 1756-0500-3-52-S2.PDF]

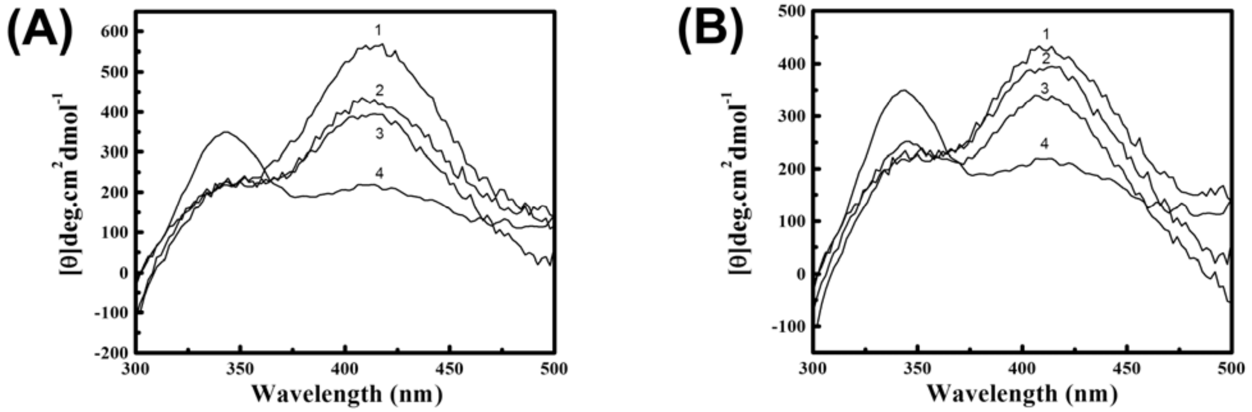

Figure: Near UV-CD spectra of EhPSAT in absence (Panel A) and presence (Panel B) of 200 mM NaCl. In both the panels the curves 1-4 represent protein samples incubated at pH 6, 7, 8 and 9 respectively.
